# WHO FCTC as a Pioneering and Learning Instrument: Comment on "The Legal Strength of International Health Instruments - What It Brings to Global Health Governance?"

**DOI:** 10.15171/ijhpm.2017.63

**Published:** 2017-05-23

**Authors:** Pekka Puska

**Affiliations:** ^1^ National Institute for Health and Welfare (THL), Helsinki, Finland.; ^2^ National Public Health Institutes (IANPHI), Helsinki, Finland.; ^3^ National Parliament, Helsinki, Finland.

**Keywords:** Tobacco Control, International Treaty, Impact Assessment, Globalization

## Abstract

The World Health Organization (WHO) Framework Convention on Tobacco Control (FCTC) is a unique global health instrument, since it is in the health field the only instrument that is international law. After the 10 years of its existence an Independent Expert Group assessed the impact of the FCTC using all available data and visiting a number of countries interviewing different stakeholders. It is quite clear that the Treaty has acted as a strong catalyst and framework for national actions and that remarkable progress in global tobacco control can be seen. At the same time FCTC has moved tobacco control in countries from a pure health issue to a legal responsibility of the whole government, and on the international level created stronger interagency collaboration. The assessment also showed the many challenges. The spread of tobacco use, as well as of other risk lifestyles, is related to globalization. FCTC is a pioneering example of global action to counteract the negative social consequences of globalization. A convention is not an easy instrument, but the FCTC has undoubtedly sparked thinking and development of other stronger public health instruments and of needed governance structures.

Haik Nikogosian and Ilona Kickbusch discuss the role of the World Health Organization (WHO) Framework Convention on Tobacco Control (FCTC) from the point of view of legal international health instruments in their article.^1^ The topic is of great importance. Health is becoming increasingly important on global arena – not only as health issue but also concerning its impact on socio-economic and sustainable development.

This has been witnessed both in the increasing attention to health crises, such as SARS, bird flu and Ebola, and in attention to the contemporary heavy burden of noncommunicable diseases (NCDs) on global health mechanism for international infectious disease control mechanism WHO has a long time had the International Health Regulations (IHR) as binding instrument and control. The problems and shortcomings of this instrument have during the last few years been discussed in relation to lessons learned from the recent epidemics. The compliance by the Member States has been of particular concern.

The emergence of NCDs as the leading public health burden has brought new type of demands for both international collaboration and national actions. This time the question is not of microbes but of lifestyles and their determinants.^2^ It means that the needed actions go far beyond health care and even health policies – to intersectoral activities and Health in All Policies.^3^

WHO has responded to the challenges of NCDs by its Global NCD Strategy and the Global NCD Action Plan 2013-2020.^4^ This Action Plan is the response to the High Level Political Declaration of United Nations (UN) in 2011. The UN Political Declaration and the WHO Action Plan with concrete targets outline well the needed international and national actions. But the problem lies in the implementation. It has often been said that public health would hugely improve, if even a part of the recommended actions would take place. Thus the big problem is the “implementation gap” and the health governance.

This kind of discussion has drawn attention to the nature of international and global health instruments. All the good strategies and programmes of WHO are only recommendations to the Member States. Obviously they are endorsed by ministers of health. But what about the governments as a whole? There is no binding nature. In this respect the FCTC is different and pioneering. It is binding for the countries that have ratified the Convention.

In this discussion the pioneering nature of the WHO FCTC is drawing increasing attention.^5^ Nikogosian and Kickbusch discuss the background, principles and role of the FCTC. The Convention grew from the devastating role of tobacco use on global public health and from the very strong medical evidence.^6^ Cigarettes are unique commercial products that kill – and even in mass scale – when used as intended. Currently 181 countries are Parties of the FCTC.

Since the FCTC has now been in force for over 10 years, we have good opportunity to learn from the experience – not only for global tobacco control but also more broadly as a global health instrument. At the sixth biannual meeting of the Conference of Parties of the FCTC (COP6) in 2014 it was decided that after 10 years of the FCTC an overall impact assessment should be carried out and that an independent group of experts should undertake the task. The Group was set up and it carried out the work in 2015-2016. The Expert Group presented its report to the Seventh Meeting of the COP in November in New Delhi, India.^7^

The Group reviewed the reports of the FCTC member countries (“Parties”). The reports show big variation in the implementation rates of the different FCTC articles.^8^ According to those reports the best implemented articles are those that concern smokefree areas (art. 8), labeling of tobacco products (art. 11) and sale to minors (art. 16). The Group reviewed also the results of the International Tobacco Control Policy Evaluation Project, the ITC Project concerning the global evidence on the implementation and effectiveness of the tobacco control measures of the FCTC.^9^

The results of the ITC Project showed more specifically the extent of implementation and the role of the FCTC concerning different articles. The conclusion was that the FCTC has contributed to significant and rapid progress for articles on smokefree areas, on labeling, on education and communication, on sale to minors and on reporting. The results further showed that the FCTC has contributed to progress with articles on price and tax, on product disclosures, on tobacco advertising and promotion, on tobacco cessation, on illicit trade and on research and surveillance. It was also concluded that the FCTC has brought widespread awareness and compliance to the article 5.3. on avoiding industry interference.

To understand more how this kind of international convention works and has an influence on tobacco control policies and actions in countries the Expert Group visited twelve countries form different regions of the world and from different economic levels. During the visits the Group interviewed a large number of different stakeholders about the process of tobacco control and the role of the of the FCTC. It was quite clear that the FCTC has played a pivotal role in countries where effective tobacco control was not in place before the FCTC and helped to strengthen policies in countries where this was the case before the ratification. Particularly for the developing world countries their participation in the preparation negotiations has brought not only detailed understanding of the articles but also “ownership” of the Convention. At the same time the Group realized the numerous gaps and shortcomings in the practical implementation, as mentioned earlier in the “implementation gap” discussion.

The Expert Group country missions found strong affirmation of the importance and use of the FCTC. The Convention has clearly acted both as a catalyst for tobacco control and, together with the guidelines, as a framework for action. At the same time the Group realized how international tobacco industry has intensified it opposition, and with prohibition of advertising and promotion, by using several other strategies. While ministries of health generally resist the industry pressures, some other parts of the government, like ministries of finance, have more often industry interference.

A very important observation is that FCTC being a binding instrument, usually ratified by the Parliament, tobacco control has become not only a health issue and a function of the ministry of health, but also an administrative and legal responsibility of the different sectors of the government. Many countries have created multisectoral structures for the work. The implementation of the Convention has also formal recognition of non-governmental organization (NGO) coalitions and has provided a basis to press the government to act. More generally, the role of NGOs was important in creating the FCTC, and is continuously important for its implementation both internationally and within the countries.

The McCabe Centre in Australia (http://www.mccabecentre.org) as a knowledge hub of the FCTC reviewed for the Group the international experience concerning the role of the Convention as an international legal instrument in protecting national tobacco control measures from legal challenges brought against such measures by tobacco industry. Review of a number of court cases from different countries have shown how the FCTC has guided the courts and provided legal basis for decisions to favour the tobacco control legislation.

It is also clear that the Convention has had an impact on a range of global and governance institutions and agendas, especially concerning the global NCD agenda and the UN 2030 Sustainable Development Agenda. The Convention has been the basis for the engagement of other UN system members in tobacco control through UN Interagency Task Force, through collaborative initiatives with the United Nations Office on Drugs and Crime (UNODC) and with The United Nations Development Programme (UNDP) to promote national development strategies including tobacco control.

Thus overall, it is quite obvious that the years of the FCTC have seen some remarkable development in global tobacco control. The Expert Group received also information from the countries with available data that there was an overall decline in smoking prevalence between 2005 and 2015 and that countries with stronger implementation of the FCTC articles had significantly greater decline in smoking.^10^

It will never be possible to precisely assess how much the measures are directly or indirectly attributable to the Convention. But it is clear that the FCTC has both on national and international level acted as spearhead, strong catalyst and roadmap for strengthening the NCD agenda and action. At the same time stronger political and technical support to low- and middle-income countries is needed, especially to counteract the strong opposition and pressures from the tobacco industry.

Global work on tobacco control, as with NCD related lifestyles in general, is closely linked to issues of globalization. The International Labour Organization (ILO) Commission on “Social consequences of Globalization” stated in its report in 2004 that globalization has, in addition to many favourable impacts, also negative effects on people’s health and environment. The Commission recommended that international community should take action to counteract these negative consequences. Tobacco epidemic is a good example, and the FCTC is a pioneering and good example on actions to start to counteract the global problem.^11^

An interesting and difficult question is whether there is time and case for other health related conventions. In the area of environmental protection much progress is taking place due to conventions. As far as health is concerned, alcohol and some aspects of diet/food have sometimes been mentioned as topics for further conventions. The evaluation of the FCTC gives us great insight to the many aspects of a health related convention. While there is little doubt on the great role and impact of the FCTC, a convention is not an easy instrument – especially in the health field. This is because of the multidimensional nature of diet (unlike tobacco) and the numerous stakeholders with conflicting and strong interests. While these discussions continue it is important, in addition to ensuring the full implementation of the FCTC, to learn about the many lessons with the FCTC and also to develop other type of global instruments and the global health coverage.

## Ethical issues

Not applicable.

## Competing interests

Author declares that he has no competing interests.

## Author’s contribution

PP is the single author of the paper.
